# Burnout and depression in the UK dental workforce: findings from a cross-sectional survey

**DOI:** 10.1038/s41415-025-8605-7

**Published:** 2025-08-08

**Authors:** Jennifer Knights, Linda Young, Gerry Humphris, Tim Newton, UK Dental Team Mental Health Research and Implementation Group

**Affiliations:** 064963848402698224989 NHS Education for Scotland, Edinburgh, UK; University of Dundee, Dundee, United Kingdom; 206639837335303348138 NHS Education for Scotland, Edinburgh, United Kingdom; 075597331098446685734 University of St Andrews, St Andrews, United Kingdom; 580301840314545494386 King´s College London, London, United Kingdom

## Abstract

**Introduction** As the crisis in UK dentistry shows few signs of abating, there is a need for ongoing assessment of the mental health of all members of the dental team and development of the evidence base needed for effective interventions.

**Aim** The aim of this paper was to evaluate current levels of burnout, depressed mood, experienced trauma and preparedness to provide quality care in dental teams in the UK.

**Methods** An anonymous cross-sectional survey was undertaken in 2023 of all members of the dental workforce, across the four constituent countries of the UK. In total, 1,507 members of the dental workforce completed the questionnaire. Quantitative data were analysed using Stata/MP 16.1 and subjected to descriptive calculations, analysis of variance and multiple regression analysis.

**Results** Dental staff reported high levels of negative psychosocial impact, including depressive symptomology, burnout and trauma. In total, 61% of respondents scored as ‘high' on the emotional exhaustion scale, with dentists in particular suffering on this measure. High levels of respondents were also experiencing feelings of detachment towards patients. Feelings of low personal accomplishment appeared to be most pronounced in dental care professionals. Participants with an NHS commitment between 75-99% were experiencing significantly higher levels of emotional exhaustion and depressive symptomology and lower levels of personal accomplishment. Self-employed participants had significantly higher levels of emotional exhaustion and depersonalisation than employed participants. Age was a significant predictor across all measures (except post-traumatic stress disorder), with early-mid career participants found to be in particular difficulty. Overall, 8% of respondents were exhibiting signs of occupational burnout, rising to 10% for dentists alone. The proportion of respondents reporting symptoms suggestive of a diagnosis of depression was over 36%. While depressive symptomology was lower among practice managers and receptionists than dentists and dental care professionals, for all professional groups, it was still far in excess of what would be expected in a population-based cohort. On average, all respondents felt prepared to provide quality practice; although, dentists felt less prepared. One-quarter of participants reported an ongoing traumatic impact from the COVID-19 pandemic.

**Conclusion** A sizeable group of people working in dental care settings in the UK are experiencing psychological distress. Solutions require change at the system level, identifying and modifying those conditions that create an environment that promotes burnout and depression. There is an urgent need for all four countries of the UK to reform the system within which NHS dentistry is delivered to improve the psychological safety of the whole dental team.

**Supplementary Information:**

Zusatzmaterial online: Zu diesem Beitrag sind unter 10.1038/s41415-025-8605-7 für autorisierte Leser zusätzliche Dateien abrufbar.

## Key points

- There is a sizeable group of people working in dental care settings in the UK who are experiencing psychological distress, including depressive symptomology, burnout and trauma.
- Findings in this study suggest that the situation has not improved since the COVID-19 pandemic and in fact may even have worsened.
- Solutions require change at the system level, identifying and modifying those conditions that create an environment that promotes burnout and depression.

**Supplementary Information:**

Zusatzmaterial online: Zu diesem Beitrag sind unter 10.1038/s41415-025-8605-7 für autorisierte Leser zusätzliche Dateien abrufbar.

## Introduction

Dentistry in the United Kingdom (UK) is in crisis. The current situation might be best understood as constituting a ‘wicked problem', characterised as having no definitive description, being without precedent and consisting of numerous and complex causes.^1^^,^^2^ Focusing on NHS dentistry in England, the Nuffield Trust identify multifarious issues, including persistent and growing inequality of access and outcomes, wide variations in treatment between regions, contractual inadequacy, and severe underspending on dentistry by the NHS (National Health Service).^3^ The NHS Dental Recovery Plan 2024 (England) comprehensively failed to deliver its target of 1.5 million additional courses of treatment in 2024-25.^4^ Public perceptions of access and cost have become a serious issue, with satisfaction in NHS dentistry services at a record low of 24%.^5^

In terms of the dental workforce, steps to increase training places in the dental professions and redistribute dental activity among different clinical roles have been outlined as urgent and necessary to meet future supply and demand needs.^3^^,^^6^ Yet, the success of these policies is inevitably dependant on the attractiveness of the dental professions as a set of occupations and the ability of the system to retain staff. A review of the recent literature relating to staff wellbeing, however, brings the ability of UK dentistry to maintain and retain a productive and effective workforce into serious question. During the COVID-19 pandemic, studies investigating the mental health and wellbeing of clinical dental staff in the UK consistently reported the negative impact of the pandemic on stress, anxiety and burnout in dentistry.^7^ A longitudinal investigation in Scotland of those working in primary care dental teams found 27% of participants were reporting depressive symptoms (compared with 18% in a population-based cohort) and 55% were experiencing emotional exhaustion.^8^ Weekly diaries completed by a sub-set of the sample showed, on average, a 25% deterioration in wellbeing between July and December 2020.^9^ The investigation also identified significant concerns surrounding the sustainability of dental services and dental training programmes in the medium- to longer-term.^10^ A survey in the South West of England found dental therapists and hygienists were experiencing low levels of mental wellbeing compared to the general population and 45% of respondents reported high anxiety levels.^11^ Another survey found three-quarters of dentists in Wales had gone to work despite not feeling mentally well enough.^12^

There is a long-standing association between dentistry and poor mental health.^13^ However, evidence collected during the pandemic highlighted an increased vulnerability of all members of the dental team to the development of burnout and other impacts from occupational stress. As the crisis in UK dentistry shows few signs of abating, there is a responsibility upon the dental research community to continue to assess the experiences of the dental workforce and further develop the evidence base needed for effective interventions to improve mental health in dentistry.^7^ In 2022, the UK Dental Team Mental Health Research and Implementation Group was formed, comprising experts in mental health and dentistry from the four UK countries (England and the devolved countries: Scotland, Wales and Northern Ireland).^14^ The group initiated the Mental health IN Dental SETtings U.K. Project (MINDSET U.K.) to evaluate current levels of burnout, depressed mood, experienced trauma and preparedness to provide quality care in dental teams in the UK. In this paper, we present the quantitative findings from the MINDSET U.K. Survey 2023.

## Methods

### Study design

The study comprised an anonymous cross-sectional survey of all members of the dental workforce, across the four constituent countries of the UK. Professional groupings in this workforce include dentists, dental care professionals (DCPs: dental nurses; dental hygienists; dental therapists; orthodontic therapists; dental technicians; clinical dental technicians) and administrative staff (AS: dental practice managers; dental receptionists). Settings included the General Dental Service (GDS), both independent and corporate, Public/Community Dental Service (PDS/CDS), hospital dental service, armed forces, prison service, public health and higher education institutions.

There are no UK-wide registers of contact details for members of these professional groups who are currently working in NHS dental services. Respondents were therefore invited via country-specific recruitment strategies overseen by the chief investigator for each country. Primary strategies were as follows: in England, dissemination was coordinated via a wide range of organisations, associations and specialist dental societies; in Wales, the survey was shared via distribution lists held by Health Education and Improvement Wales (HEIW); in Northern Ireland, distribution was taken forward through Health and Social Care Northern Ireland; and in Scotland, invitations were distributed using NHS Education for Scotland's (NES) Portal, an online course booking system where dental professionals who use NES' services can opt in to receive marketing communications. The questionnaire was open from 19 April 2023 to 30 June 2023 and hosted on Microsoft Forms by NES.

### Questionnaire

In addition to demographic information and a free-text box for comments, the questionnaire gathered information about participants' mental health and wellbeing using the following six health and wellbeing scales:

- The three sub-scales of the Maslach Burnout Inventory: emotional exhaustion (EE); depersonalisation (DP); and personal accomplishment (PA). Participants were asked to answer the items in each scale in relation to their current role. Response options ranged from ‘never', coded 0, to ‘every day', coded 6. Responses were summed to give sub-scale scores. For the EE and DP sub-scales, a higher summed score is indicative of a higher risk of burnout, whereas for the PA, sub-scale scoring is reversed. Published thresholds were used to define high EE (≥27), high DP (≥13) and low PA (≤27). Burnout was defined as high EE plus high DP plus low PA
- The Patient Health Questionnaire-2 (PHQ-2): a two-item scale used to screen for depressive symptomology. Participants were asked to answer both items in respect of their feelings during the previous two weeks. Response options ranged from ‘not at all', coded 0, to ‘nearly every day', coded 3, and were summed to give an overall score (0-6). A summed score ≥3 was defined as indicative of depressive symptomology
- The Impact of Event Scale-6 (IES-6): a shortened version of 22-item IES-Revised was used to screen for post-traumatic stress disorder (PTSD). Participants were asked to indicate how distressing the difficulty described by each item had been for them in the previous seven days. At the time of the survey, it was hypothesised that NHS dental services were still recovering from the impact of the COVID-19 pandemic and, therefore, participants were asked to answer with reference to COVID-19. Response options ranged from ‘not at all', coded 0, to ‘extremely', coded 4. The overall score was calculated as the mean of the six items with a mean score ≥1.75 considered to be indicative of PTSD
- The Dental Preparedness for Quality Practice Scale (DPQPS): comprised seven items, derived from the P-Qual-C19 sub-scale which was used to measure NHS primary care dental teams' and trainees' preparedness to provide quality care during the COVID-19 pandemic.^7^ In this study, the stem of the scale did not reference COVID-19 but simply asked participants how well-prepared they felt in their current role. Response options ranged from ‘extremely well-prepared', coded 1, to ‘unprepared', coded 5. The overall score was calculated as the mean of the seven items with a lower mean score indicating greater preparedness.

These widely used measurement scales relate to mental fatigue and stress level. They do not conform to measures from which DSM-5-TR (Diagnostic and Statistical Manual of Mental Disorders) or ICD-11 (International Classification of Diseases) codes of disease category can be derived and are not formal measures of health status. Instead, they provide hypothetical points along some dimensions of wellbeing that are meaningful within a survey context to assist with predictions and identification of associations with environmental factors in the workplace. A copy of the questionnaire is available in the online Supplementary Information.

### Sample sizes

The study included the four countries within the UK. Hence, we estimated a minimum sample for testing comparisons within each country. A sample of 375 is indicated when fitting a linear model where two covariates are entered (age and sex) which would account for more than 3% of the variance in the dependent variable (e.g., depression rating) and would detect a further 2% of the dependent variable variance attributed to entry of the factor under test (e.g., staff grouping), under the condition of 80% power and a conventional alpha level of 0.05.

### Data analysis

Analysis of quantitative data was carried out in Stata/MP 16.1. Descriptive global and subgroup statistics by professional group are presented for all demographic items and health and wellbeing scales. Demographic descriptives detail the number and percentage of participants in each response category. Percentages include valid responses only. Totals may not sum to 100 due to rounding. For each health and wellbeing scale, the mean score and standard deviation are presented as the measures of central tendency. In addition, the total scores of those measures were compared to published threshold values where available. Missing data from individual items within scales (unless greater than 50% of items comprising a scale) were pro-rated to provide estimated values.

Analysis of variance was used as the omnibus test to compare health and wellbeing scale means between professional groups. Given unequal group sizes, where the group variances were heterogeneous (Bartlett's test chi-square <0.05), the Welch F statistic is reported. Post-hoc tests were carried out when the omnibus test was statistically significant using the Bonferroni or Games and Howell adjustment as appropriate. Associations between individual health and wellbeing scales and participant demographics were explored using multiple regression analysis. Robust regression was used when the variance of the errors was heterogeneous. For all statistical tests, the *a priori* criterion for statistical significance was set at p <0.05.

### Ethics, governance and data protection

Following assessment of key characteristics against the NHS Health Research Authority's ‘defining research' table,^15^ the study was classified as an evaluation of service impact on service delivery staff and therefore did not require NHS Research and Development review and approval or institutional review. This outcome was confirmed by the following bodies: NES, NHS Tayside, HEIW, Kings College London, and the Office of Research Ethics Northern Ireland.

A participant information sheet (PIS) was provided, containing detailed information about the study. At the outset of the questionnaire, respondents were asked to confirm they had read and understood the PIS and understood that the data collected was anonymous. A positive response to both questions and completion of the questionnaire implied consent. The PIS signposted respondents to their doctor or NHS Practitioner Health should they wish to speak with someone about their mental health. A weblink to various charitable helplines was also provided.

### Role of the funding source

Each member of the study team was funded through core institutional budgets. The funders of the study had no role in study design, data collection, data analysis, data interpretation, or writing of the manuscript.

## Results

### Participants and demographic descriptives

In total, 1,538 questionnaires were submitted, where 31 submissions contained no data, leaving a survey cohort comprising of 1,507 members of the dental workforce. The cohort included members of the dental workforce from all three professional groups (dentists = 59%; DCPs = 32%; AS = 9%) and all four UK countries (England = 28%; Northern Ireland = 13%; Scotland = 48%; Wales = 11%). Most participants worked in either the GDS (67%) or the PDS/CDS (18%), with a minority (16%) working in other settings. Just over half (52%) were employed, with the remaining 48% being self-employed and 83% providing direct clinical care. The majority of participants (58%) provided both NHS and private services; 30% only provided NHS services and 9% only provided private services. Additionally, 4% did not know what types of services were provided in their workplace. The demographic characteristics of the participants are summarised in Table 1.

**Table 1:** Description of the demographic characteristics of respondents (*n* = 1,507)

**Variable**		**Dentists**	**DCPs**	**AS**	**Total sample**
		**n (%)**	**n (%)**	**n (%)**	**n (%)**
Country	England	245 (27.8)	148 (30.6)	25 (19.2)	418 (28.0)
	Northern Ireland	140 (15.9)	38 (7.9)	19 (14.6)	197 (13.2)
	Scotland	404 (45.8)	239 (49.4)	70 (53.9)	713 (47.6)
	Wales	90 (10.2)	59 (12.2)	16 (12.1)	165 (11.0)
	Other	4 (0.5)	0 (0.0)	0 (0.0)	4 (0.3)
Sex	Male	341 (38.5)	24 (5.0)	5 (3.9)	370 (24.7)
	Female	524 (59.2)	456 (94.0)	124 (95.4)	1,104 (73.6)
	Prefer not to say	20 (2.3)	5 (1.0)	1 (0.8)	26 (1.7)
	Identify in another way	0 (0.0)	0 (0.0)	0 (0.0)	0 (0.0)
Age (years)	18-34	199 (22.5)	107 (22.1)	19 (14.6)	295 (19.7)
	35-44	247 (27.9)	133 (27.4)	38 (29.2)	418 (27.9)
	45-54	243 (27.5)	138 (28.5)	44 (33.9)	425 (28.3)
	55+	185 (20.9)	102 (21.0)	28 (21.5)	287 (19.1)
	Prefer not to say	11 (1.2)	5 (1.0)	1 (0.8)	17 (1.1)
Work setting	GDS independent	504 (57.1)	184 (38.5)	70 (54.3)	758 (50.9)
	GDS corporate	160 (18.1)	60 (12.6)	20 (15.5)	240 (16.1)
	CDS/PDS	132 (15.0)	112 (23.4)	15 (11.6)	259 (17.4)
	Public health	3 (0.3)	33 (6.9)	15 (11.6)	51 (3.4)
	Hospital	60 (6.8)	43 (9.0)	6 (4.7)	109 (7.3)
	Other	23 (2.6)	46 (9.6)	3 (2.3)	72 (4.8)
NHS commitment	Fully NHS	225 (25.5)	185 (38.5)	36 (27.7)	446 (29.9)
	Mostly NHS	465 (52.7)	136 (28.3)	56 (43.1)	657 (44.0)
	Mostly private	124 (14.0)	63 (13.1)	22 (16.9)	209 (14.0)
	Fully private	63 (7.1)	58 (12.1)	9 (6.9)	130 (8.7)
	Don't know	6 (0.7)	39 (8.1)	7 (5.4)	52 (3.5)
Clinical care	Yes	852 (97.3)	358 (74.3)	26 (20.0)	1236 (83.1)
	No	24 (2.7)	124 (25.7)	104 (80.0)	252 (16.9)
Employment	Self-employed	650 (73.9)	66 (13.7)	2 (1.6)	718 (48.2)
	Employed	230 (26.1)	417 (86.3)	126 (98.4)	773 (51.8)

### Main results (mental health and wellbeing scales)

Table 2 summarises the descriptive scores and group comparisons for all mental health and wellbeing scales by professional group. Notably, 60.8% of participants reported signs of emotional exhaustion (EE score ≥27). Examination of scores from the PHQ-2 and IES-6 scales found that signs of depressive symptomology were exhibited by 36% of participants and signs of PTSD from the ongoing impacts of the COVID-19 pandemic by 25% of participants. There is no published threshold for the DPQPS measure, but on average, participants reported they felt prepared to provide quality care (mean = 2.2; SD = 0.7).

**Table 2:** Psychometric scales and levels of burnout shown by job role

**Psychometric scale**	**Dentists**	**DCPs**	**AS**	**Total**	**Omnibus**		**Dentist versus DCPs**		**Dentist versus AS**		**DCP versus AS**	
	**Mean (SD)**	**Mean (SD)**	**Mean (SD)**	**Mean (SD)**	**F (DF)**	**P**	**Difference**	**P**	**Difference**	**P**	**Difference**	**P**
Emotional exhaustion (EE)	34.2 (15.0)	26.7 (15.6)	25.7 (15.4)	31.0 (15.7)	46.2 (2, 1,496)	<0.01	7.5	<0.01	8.5	<0.01	1.0	>0.99
n (%) above cut-off	618 (69.8)	240 (49.6)	53 (40.8)	911 (60.8)								
Depersonalisation (DP)	9.8 (7.7)	5.4 (6.0)	6.7 (6.8)	8.1 (7.4)	69.3* (2, 360.3)	<0.01	4.4	<0.05	3.1	<0.05	-1.3	≥0.05
n (%) above cut-off	273 (30.9)	63 (13.0)	28 (21.5)	364 (24.3)								
Personal accomplishment (PA)	32.2 (7.8)	29.6 (10.0)	30.7 (9.5)	31.2 (8.8)	12.7* (2, 332.2)	<0.01	2.6	<0.05	1.5	≥0.05	-1.1	≥0.05
n (%) below cut-off	200 (22.6)	159 (32.8)	41 (31.5)	400 (26.7)								
Overall burnout (+ve EE, +ve DP and -ve PA) n (%)	88 (10.0)	21 (4.4)	10 (7.7)	119 (8.0)								
Depressive symptomology (PHQ2)	2.4 (2.0)	2.3 (1.9)	2.0 (1.9)	2.3 (1.9)	3.8 (2, 1,494)	0.02	0.2	0.34	0.5	0.03	0.3	0.39
n (%) above cut-off	336 (38.1)	172 (35.5)	36 (27.7)	544 (36.3)								
Impact of events (IES)	1.1 (1.1)	1.1 (1.0)	1.1 (1.0)	1.1 (1.0)	0.09 (2, 1,493)	0.9						
n (%) above cut-off												
Preparedness for quality practice (DPQPS)	2.3 (0.7)	2.0 (0.7)	1.9 (0.7)	2.2 (0.7)	39.44 (2, 1,494)		0.3	<0.01	0.4	<0.01	0.1	0.4
Key: * = Welch F statistic												

Omnibus testing found no statistically significant difference between the professional groups when comparing IES-6 scores (f = 0.09, p = 0.91). For all other mental health and wellbeing scales, omnibus tests found significant between group differences (p <0.05). Examination of post-hoc results did not identify a statistically significant difference between the DCP and AS groups in any scale.

In the burnout scales, DCPs' EE scores were significantly lower than dentists‘ (diff = -7.46, 95% CI [-9.52, -5.39]; p <0.01) as were the scores of the AS group (diff = -8.48, 95% CI [-11.91, -5.05]; p <0.01). The levels of depersonalisation exhibited by the DCP and AS groups were also significantly lower than those exhibited by dentists (DCP diff = -4.38, 95% CI [-5.23, -3.50]; p <0.05: AS diff = -3.08, 95% CI [-4.62, -1.54]; p <0.05). Levels of personal accomplishment were significantly higher in the dentist group than in the DCP group (diff = -2.58, 95% CI [-3.81, -1.3]; p <0.05) whereas the difference between the dentist and AS groups was not significant.

Levels of depressive symptomology were significantly lower in the AS group than in the dentist group (diff = -0.46, 95% CI [-0.89, -0.03]; p = 0.03). The difference between the DCP and dentist groups was not significant. On average, all groups felt prepared to provide quality care. However, compared to the dentist group, feelings of preparedness were significantly higher in the DCP group (diff = -0.30, 95% CI [-0.39, -0.20]; p <0.01) and in the AS group (diff = -0.40, 95% CI [-0.56, -0.25]; p <0.01).

Multiple linear regression of participants' demographic characteristics (professional group, age, sex, country, NHS commitment and employment status) against each of the individual mental health and wellbeing scales was used to explore associations between these variables (Table 3). Due to violation of the linear regression homoscedasticity assumption, robust regression was used for the EE, DP, PA and IES models.

**Table 3:** Associations between demographic characteristics and psychometric scales

**Variable**		**EE** **F(16, 1,381) = 13.71**	**DP** **F(16, 1,381) = 19.11**	**PA** **F(16, 1,380) = 5.79**	**PHQ-2** **F(16, 1,380) = 6.23**	**IES-6** **F(16, 1,379) = 1.88**	**DPQPS** **F(16, 379) = 6.15**
		**β (95% CI: P)**	**β (95% CI: P)**	**β (95% CI: P)**	**β (95% CI: P)**	**β (95% CI: P)**	**β (95% CI: P)**
Prof group	Ref: dentist						
	DCP	-3.46 (-5.89, -1.03: 0.01)	-2.12 (-3.12, 1.12: <0.01)	-2.54 (-3.79, -1.29: <0.01)	0.27 (-0.02, 0.56: 0.07)	0.09 (-0.07, 0.35:0.28)	-0.22 (-0.33, -0.11: <0.01)
	AS	-4.93 (-8.61, -1.25: 0.01)	-0.74 (-2.25, 0.78: 0.34)	-1.20 (-3.09, 0.70: 0.22)	0.06 (-0.38, 0.50: 0.78)	0.12 (-0.12, 0.37: 0.33)	-0.31 (-0.47, -0.15: <0.01)
Age (years)	18-24	-0.76 (-7.12, 5.61: 0.82)	2.68 (0.07, 5.30: 0.05)	-0.03 (-3.30, 3.24: 0.99)	0.05 (-0.71, 0.81: 0.90)	-0.19 (-0.62, 0.23: 0.37)	-0.08 (-0.35, 0.20: 0.60)
	25-34	5.88 (3.23, 8.53: <0.01)	3.88 (2.79, 4.97: <0.01)	-1.25 (-2.62, 0.11: 0.07)	0.65 (0.34, 0.97: <0.001)	-0.09 (-0.27, 0.08: 0.30)	0.11 (-0.01, 0.22: 0.07)
	35-44	7.06 (4.68, 9.44: <0.01)	2.58 (1.60, 3.60: <0.01)	-1.89 (-3.12, -0.67: <0.01)	0.71 (0.43, 1.00: <0.01)	-0.002 (-0.16, 0.16: 0.98)	0.13 (0.03, 0.24: 0.01)
	45-54	5.35 (2.98, 7.71: <0.01)	0.51 (-0.46, 1.48: 0.31)	-0.96 (-2.18, 0.25: 0.12)	0.43 (0.15, 0.71: <0.01)	0.07 (-0.09, 0.22: 0.39)	0.05 (-0.06, 0.15: 0.38)
	Ref: 55+						
Sex	Ref: male						
	Female	-2.05 (-4.13, 0.04: 0.05)	-2.18 (-3.03, -1.32: <0.01)	1.11 (0.03, 2.18: 0.04)	-0.55 (-0.80, -0.30: <0.01)	-0.12 (-0.26, 0.02: 0.10)	-0.04 (-0.13, 0.05: 0.39)
Country	Ref: England						
	Northern Ireland	-0.93 (-3.72, 1.86: 0.51)	0.49 (-0.66, 1.54: 0.40)	-1.22 (-2.66, 0.21: 0.10)	-0.29 (-0.62, 0.04: 0.09)	0.16 (-0.03, 0.34: 0.10)	0.004 (-0.12, 0.13: 0.95)
	Scotland	-2.08 (-4.17, 0.01: 0.05)	0.79 (-1.65, 0.07: 0.07)	-0.49 (-1.57, 0.59: 0.37)	-0.14 (-0.40, 0.11: 0.26)	0.08 (-0.06, 0.22: 0.27)	-0.01 (-0.11, 0.08: 0.76)
	Wales	-0.45 (-3.42, 2.51: 0.77)	0.74 (-0.48, 1.96: 0.23)	-0.46 (-1.99, 1.07: 0.56)	-0.08 (-0.44, 0.27: 0.65)	0.10 (-0.10, 0.30: 0.33)	-0.02 (-0.15, 0.11: 0.78)
NHS commitment	Ref: fully NHS						
	75-99%	5.07 (3.42, 7.73: <0.01)	2.16 (1.07, 3.25: <0.01)	-3.78 (-5.15, -2.42: <0.01)	0.48 (0.16, 0.80: <0.01)	0.14 (-0.04, 0.32: 0.12)	0.02 (-0.10, 0.13: 0.78)
	50-74%	0.72 (-2.33, 3.78: 0.64)	0.28 (-0.98, 1.53: 0.67)	-1.90 (-3.47, -0.32: 0.02)	0.11 (-0.25, 0.48: 0.55)	-0.06 (-0.26, 0.14: 0.55)	-0.05 (-0.18, 0.09: 0.48)
	25-49%	-1.53 (-5.25, 2.19: 0.42)	-1.32 (-2.85, 0.21: 0.09)	-0.27 (-2.18, 1.64: 0.78)	-0.34 (-0.78, 0.11: 0.14)	-0.23 (-0.48, 0.02: 0.07)	-0.20 (-0.36, -0.03: 0.02)
	1 = 24%	-2.27 (-5.98, 1.43: 0.23)	-1.21 (-2.74, 0.31: 0.12)	-0.23 (-2.14, 1.68: 0.81)	-0.22 (-0.67, 0.22: 0.33)	-0.24 (-0.48, 0.01: 0.06)	-0.21 (-0.37, -0.04: 0.01)
	Fully private	-1.12 (-4.80, 2.55: 0.55)	- 0.95 (-2.46, 0.57: 0.22)	0.51 (-1.39, 2.40: 0.60)	0.03 (-0.41, 0.47: 0.90)	-0.04 (-0.29, 0.20: 0.73)	-0.16 (-0.32, 0.01: 0.06)
Employment	Ref: employed						
	Self-employed	4.48 (1.69, 7.26: <0.01)	1.18 (0.03, 2.33: 0.04)	1.02 (-0.41, 2.45: 0.16)	0.32 (-0.02, 0.65: 0.06)	0.05 (-0.14, 0.23: 0.62)	0.06 (-0.06, 0.18:0.33)

Relative to dentists, the models predicted that participants in the DCP professional group have significantly lower levels of EE (β = -3.46; p = 0.005), DP (β = -2.12; p = 0.001), PA (β = -2.54; p <0.001) and DPQPS (β = -0.22; p <0.001). For the AS professional group, levels of EE (β = -4.93; p = 0.009) are predicted to be significantly lower than dentist levels and preparedness for quality practice (DPQPS) (β = -0.31, p <0.001) predicted to be significantly higher than dentists' levels.

Age was a significant predictor of EE, DP, PA, depressive symptomology and preparedness for quality practice. When compared to participants aged 55 years and older, participants aged 18-24 years of age had significantly higher levels of DP (β = -2.68; p = 0.045). Those aged 25-34 years of age had significantly higher levels of EE (β = 5.88; p <0.001), DP (β = 3.88; p <0.001) and depressive symptomology (β = 0.65; p <0.001). The 35-44 years age group had significantly higher levels of EE (β = 7.06; p <0.001), DP (β = 2.58; p <0.001), depressive symptomology (β = 0.71; p <0.001) and lower levels of preparedness for quality practice (β = 0.13; p = 0.013) with significantly lower levels of PA (β = -1.89; p = 0.002). Participants aged 45-54 years of age had significantly higher levels of EE (β = 5.35; p <0.001) and depressive symptomology (β = 0.43; p = 0.003) than those aged 55 years of age and older. Age did not predict signs of PTSD from the ongoing impacts of the COVID-19 pandemic.

Compared to male participants, female participants had significantly lower levels of DP (β = -2.18; p <0.001) and depressive symptomology (PHQ) (β = -0.55; p <0.001) and higher levels of PA (β = 1.11; p = 0.04). Participants' country was not a significant predictor for levels of any of the health and wellbeing scales. Employment status predicted levels of EE and DP, with self-employed participants having significantly higher levels of EE (β = -4.48; p = 0.002) and DP (β = 1.18; p = 0.044) than employed participants. Using ‘fully NHS' as the reference category, participants with an NHS commitment between 75-99% had significantly higher levels of EE (β = 5.07; p <0.001), DP (β = 2.16; p <0.001), depressive symptomology (PHQ) (β = -0.48; p = 0.003) and lower levels of PA (β = -3.78; p <0.001). Participants with an NHS commitment of between 50-74% also had significantly lower levels of PA (β = -1.90; p = 0.018) than participants who were ‘fully NHS'.

## Discussion

This study aimed to evaluate current levels of burnout, depressed mood, experienced trauma and preparedness to provide quality care in dental teams in the UK. Dental staff reported high levels of negative psychosocial impact, including depressive symptomology, burnout and trauma.

Having 61% of respondents scoring as ‘high' on the emotional exhaustion scale is a particularly concerning finding. Although not directly comparable with the study in Scotland^8^ during 2020, this figure suggests that the situation has not improved and in fact may even have worsened since the pandemic. Dentists in particular indicate that they are suffering on this measure but the high scores across all groups are deeply troubling. Higher predicted levels of emotional exhaustion found with self-employed participants suggest that the financial uncertainty and individual responsibility for business success or failure may be playing a substantial part in increasing distress for some. Furthermore, the fact that participants with an NHS commitment between 75-99% were experiencing significantly higher levels of emotional exhaustion talks to the severe issues being faced in delivering NHS care at the present time in terms of increased demand and reduced capacity. In terms of age, it appears that those who are early- to mid-career (between 25-44) are experiencing higher emotional exhaustion than those in the later stages of their career or approaching retirement. That there is no significant difference in level of emotional exhaustion between countries is an important finding. In fact, country was not a predictor for any measure. This resonates with the key finding from a country comparison of the qualitative data collected in the study that, despite the different administrations and dental systems operating in each of the four UK countries, there was a high degree of commonality in the experiences and stressors described by respondents.^16^ The convergence of the quantitative and qualitative findings on this point strengthens the argument for strategies and responses which can be deployed UK-wide and encourages a UK-wide action plan rather than country-specific initiatives.

The finding that high levels of respondents across all groups were experiencing feelings of detachment towards patients is also a serious concern. Depersonalisation introduces a potential patient safety issue and could have effects in terms of patient satisfaction with dental care. A systematic review and meta-analysis of 170 studies incorporating 239,246 physicians found that burnout was associated with an increased likelihood of patient dissatisfaction with care, poor professionalism and patient safety incidents.^17^ In our study dentists were the most seriously affected. However, although the difference is not statistically significant, it is also of interest that the percentage of administrative staff experiencing feelings of detachment is higher than the percentage of DCPs. This might be explained in part by the increasing exposure of dental practice managers and receptionists to episodes of patient frustration and aggression, as identified in the qualitative data collected in the study.^18^ The high level of depersonalisation among those aged 18-24 is especially worrying. 

In terms of personal accomplishment, the statistically significant lower average score for DCPs in comparison to dentists raises questions about how to improve job satisfaction for this staff group. Feelings of low personal accomplishment also appear to be most pronounced for those in the middle of their career (age 35-44) which suggests that professional and personal pressures commonly experienced by those in this age range are potentially making these staff more vulnerable to feelings of low accomplishment. As with emotional exhaustion, the fact that participants with an NHS commitment between 75-99% were experiencing significantly lower levels of personal accomplishment likely speaks to the severe issues being faced by NHS dentistry.

The fact that participants with an NHS commitment between 75-99% were experiencing significantly higher levels of emotional exhaustion and lower levels of personal accomplishment compared to those who were fully NHS is an intriguing finding. One hypothesis to explain this could be that trying to deliver dental services under two systems (both NHS and private) is more demanding that committing solely to one or the other. Those dental staff with a more equal proportion of NHS versus private service (e.g., 50:50) may, however, be more rehearsed in working under either system. The fact that the survey is only a snapshot at a point in time is also potentially relevant. The proportion reported should not necessarily be considered to represent a fixed position but, with services in flux, staff may have been in the process of changing their proportions of delivery under each service type. Hence, any respondent who said that they were almost completely NHS but not quite may have been moving into the private sector gradually, with this proving particularly wearisome in comparison to the situation where they might have remained in the NHS system entirely.

With 8% of respondents exhibiting signs of occupational burnout, the potential negative impact of the current situation is cause for alarm, both in terms of the health of individuals and the future of dentistry in the UK, particularly NHS dentistry. For dentists alone, the burnout figure rises to 10%, meaning one in ten are operating at unsustainable stress levels which will likely result in many needing to take medium- to long-term sickness absence. The consequences of which are disastrous for realising urgent workforce supply goals in the sector, both in the immediacy but also in terms of staff retention. These findings confirm and expand upon a previous systematic review, which identified that the proportion of dental practitioners who were above cut-offs for burnout across all measures was 7%.^13^

The proportion of respondents reporting symptoms suggestive of a diagnosis of depression was over 36%. Again, although not directly comparable with the Scotland study^8^ during 2020, the figure suggests that the situation may have worsened since the pandemic. Levels of reporting of depressive symptomology were lower among those who did not have a direct clinical care role, but the percentage of practice managers and receptionists experiencing depressive symptomology was still far in excess (28%) of what would be expected in a population-based cohort (18%) (some of those who categorised themselves as administrative staff also reported that they provided direct clinical care [20%]. One explanation for this is that some DCPs have a dual role as administrative staff/DCP).

As with emotional exhaustion and personal accomplishment, participants with an NHS commitment between 75-99% were reporting significantly higher levels of symptoms suggestive of a diagnosis of depression, again suggestive that the pressures of NHS dentistry are a key stressor.

Although in general, all professional groups felt prepared to provide quality practice, dentists felt less prepared than DCPs and dental practice managers and receptionists. The high scores for dentists on emotional exhaustion and depersonalisation could be influenced by feelings of unpreparedness for some, for instance in terms of preparedness for managing General Dental Council and other governance requirements, as well as dealing with error and safety incidents. With one-quarter of participants reporting the traumatic impact of COVID-19, it is evident that a significant proportion of the workforce is yet to emotionally process and recover from the pressures, uncertainties and fears experienced by dental staff during the pandemic. This is consistent with the experiences reported by other healthcare staff in the first record produced by the ‘every story matters' team at the UK COVID-19 Inquiry, where contributors describe a lasting impact on their mental health from working in healthcare during the pandemic, with some having to change roles or stop working altogether.^19^

Solutions to the issues identified in this study require change not only in support of individual behaviour, but at the system level, identifying and modifying those conditions that create an environment which promotes burnout and depression.^20^ There is an urgent need for all four countries of the UK to reform the system within which NHS dentistry is delivered to improve the psychological safety of the whole dental team. To mitigate the immediate situation, funding is necessary to support the appointment of mental health champions to identify and direct those experiencing burnout and depression to appropriate care.^21^ Failure to act shall only deepen and extend the current workforce crisis in UK dentistry.

### Limitations

The findings reported here may, in part, reflect a degree of selection bias in terms of those who chose to complete the survey. It is possible that those experiencing the greatest distress are more likely to wish to complete a survey exploring wellbeing. Furthermore, the results cannot be said to be generalisable to the whole dental workforce in the UK as respondents were invited via country-specific recruitment strategies using local networks; not all eligible staff will have received the invitation to participate.

## Conclusion

Evidence collected during the COVID-19 pandemic highlighted an increased vulnerability of all members of the dental team to the development of burnout and other impacts from occupational stress. Findings in this study suggest that the situation has not improved and in fact may even have worsened since the pandemic, with a sizeable group of people working in dental care settings currently experiencing psychological distress. A particularly concerning finding is 61% of respondents scoring as ‘high' on the emotional exhaustion scale. With 8% of respondents exhibiting signs of occupational burnout, the potential negative impact of the current situation is cause for alarm, both in terms of the health of individuals and the future of dentistry in the UK, particularly NHS dentistry. We argue that there is an urgent need for system-level reform of NHS dentistry across all four countries of the UK to improve the psychological safety and wellbeing of the whole dental team.

## Resources

The British Dental Association has compiled a selection of resources to help support wellbeing, build resilience and connect with colleagues: https://www.bda.org/advice/wellbeing/.

## Supplementary Information

Supplementary Information (PDF 644KB)

## Data Availability

The dataset presented in this article is controlled by NHS Education for Scotland. Access to the data are subject to approval and a data sharing agreement. Access requests should be directed to sdpbrn@nes.scot.nhs.uk.
