# Supplementary material for: Burnout and depression in the UK dental workforce: findings from a cross-sectional survey
Source: Br Dent J. 2025 Aug 8;239(3):189–96. doi: 10.1038/s41415-025-8605-7 (PMC12334352; doi:10.1038/s41415-025-8605-7)
Supplement: Supplementary file 1 — Supplementary Information (PDF 644KB) [file 41415_2025_8605_MOESM1_ESM.pdf]

Mental health IN Dental SETtings U.K. Project (MINDSET U.K.)

The purpose of this questionnaire is to evaluate current levels of burnout, depressed mood, experienced trauma and preparedness to provide quality care in dental teams in the UK.

Most of the questions require you to select an answer, but there is also a text box that we hope you will use. Please do not take too long over your replies, your immediate reaction to each question is likely to be more accurate than a long thought-out response.

Be assured that the questionnaire is **anonymous**, and you cannot be identified from the answers you provide or in any report or publication that arises from this work. It should take approximately 5 to 10 [illegible]

In order for you to participate **anonymously** in this survey, please complete the questions below.

1. I have read and understood the participant information sheet that was attached to the email message

◯ Yes

◯ No

2. I understand that by being a participant in this study that the data collected is anonymous

◯ Yes

◯ No

3. Please answer each of the below questions in relation to your current role. If you do not deal with patients in your day-to-day role then please answer in respect to the staff and individuals with whom you work most regularly:

	Never	A few times a year	Once a month	A few times a month	Once a week	A few times a week	Every day
I feel emotionally drained from my work	○	○	○	○	○	○	○
I feel used up at the end of the workday	○	○	○	○	○	○	○
I feel fatigued when I get up in the morning and have to face another day on the job	○	○	○	○	○	○	○
Working with people all day is really a strain for me	○	○	○	○	○	○	○
I feel burned out from my work	○	○	○	○	○	○	○
I feel frustrated by my job	○	○	○	○	○	○	○
I feel I'm working too hard on my job	○	○	○	○	○	○	○
Working with people directly puts too much stress on me	○	○	○	○	○	○	○
I feel like I am on the end of my rope	○	○	○	○	○	○	○

4. Please answer each of the below questions in relation to your current role. If you do not deal with patients in your day-to-day role then please answer in respect to the staff and individuals with whom you work most regularly.

	Never	A few times a year	Once a month	A few times a month	Once a week	A few times a week	Every day
I can easily understand how my patients feel about things	○	○	○	○	○	○	○
I deal very effectively with the problems of my patients	○	○	○	○	○	○	○
I feel I'm positively influencing other people's lives through my work	○	○	○	○	○	○	○
I can easily create a relaxed atmosphere with my patients	○	○	○	○	○	○	○
I feel exhilarated after working closely with my patients	○	○	○	○	○	○	○
I have accomplished many worthwhile things in this job	○	○	○	○	○	○	○
In my work, I deal with emotional problems very calmly	○	○	○	○	○	○	○

5. Please answer each of the below questions in relation to your current role. If you do not deal with patients in your day-to-day role then please answer in respect to the staff and individuals with whom you work most regularly:

	Never	A few times a year	Once a month	A few times a month	Once a week	A few times a week	Every day
I feel I treat some patients as if they were impersonal 'objects'	◯	◯	◯	◯	◯	◯	◯
I've become more callous towards people since I took this job	◯	◯	◯	◯	◯	◯	◯
I worry that this job is hardening me emotionally	◯	◯	◯	◯	◯	◯	◯
I don't really care what happens to some patients	◯	◯	◯	◯	◯	◯	◯
I feel patients blame me for some of their problems	◯	◯	◯	◯	◯	◯	◯

6. Over the last 2 weeks, how often have you been bothered by any of the following problems?

	Not at all	Several days	More than half the days	Nearly every day
Little interest or pleasure in doing things	○	○	○	○
Feeling down, depressed, or hopeless	○	○	○	○

7. We appreciate that dental workplaces are still recovering from the impact of the COVID-19 pandemic. Thinking about COVID-19 please read each item, and then indicate how distressing each difficulty has been for you DURING THE PAST SEVEN DAYS with reference to your work/practice:

	Not at all	A little bit	Moderately	Quite a bit	Extremely
I thought about it when I didn't mean to	○	○	○	○	○
I felt watchful or on-guard	○	○	○	○	○
Other things kept making me think about it	○	○	○	○	○
I was aware that I still had a lot of feelings about it but I didn't deal with them	○	○	○	○	○
I tried not to think about it	○	○	○	○	○
I had trouble concentrating	○	○	○	○	○

8. In your current role how well prepared are you for:

	Extremely well prepared	Well prepared	Prepared	Not well Prepared	Unprepare d	Not applicable
Coping with GDC governance and training requirements	○	○	○	○	○	○
Maintaining good quality of care	○	○	○	○	○	○
Taking part in clinical governance	○	○	○	○	○	○
Reducing the risk of cross-infection	○	○	○	○	○	○
Organisation al decision making	○	○	○	○	○	○
Ensuring patient safety	○	○	○	○	○	○
Reporting and dealing with error and safety incidents	○	○	○	○	○	○

9. If you would like to expand upon any of your answers in this questionnaire, please do so in this box. Please do not include any information which would allow us to identify you.

10. Age

- 18-24
- 25-34
- 35-44
- 45-54
- 55-64
- 65 and over
- Prefer not to say

11. Sex

- Male
- Female
- Identify in another way
- Prefer not to say

12. Professional Group

- Dentist
- Dental Care Professional
- Practice Manager/Receptionist

13. Are you currently in training?

- ◯ Yes
- ◯ No

14. Do you provide direct clinical care?

- ◯ Yes
- ◯ No

15. Main place of work

- ◯ GDS (Independent)
- ◯ GDS (Corporate)
- ◯ Hospital
- ◯ CDS/PDS
- ◯ Armed Forces
- ◯ Prison Service
- ◯ Public Health
- ◯ Higher Education Institution
- ◯ Other

16. Employment (main place of work)

- ◯ Self Employed
- ◯ Salaried/Employed

17. NHS Percentage (main place of work)

- ◯ 100% (exclusively NHS)
- ◯ 75-99%
- ◯ 50—74%
- ◯ 25-49%
- ◯ 1-24%
- ◯ 0% (exclusively private)
- ◯ Don't know

18. Personal NHS commitment (if applicable)

- ◯ 100% (exclusively NHS)
- ◯ 75-99%
- ◯ 50—74%
- ◯ 25-49%
- ◯ 1-24%
- ◯ 0% (exclusively private)

19. Which country do you work in?

- ◯ England
- ◯ Northern Ireland
- ◯ Scotland
- ◯ Wales
- ◯ Other
